# Core sepsis-related competencies for medical students: an international consensus by Delphi technique

**DOI:** 10.1186/s12909-024-05525-9

**Published:** 2024-06-11

**Authors:** Elanor Lian Mary Gomersall, Lowell Ling, Konrad Reinhart, Victoria Bion, Abeselom Ekesh, Christiana Adu-Takyi, Luciano Cesar Pontes Azevedo, Paulin Ruhato Banguti, Jonathan Cohen, Janet Victoria Diaz, Bin Du, David M. Goldfarb, Luis Antonio Gorordo-Delsol, Colin Alexander Graham, Ricardo Iramain, Shevin T. Jacob, Zsuzsoka Kecskes, Niranjan Kissoon, Jeffrey Lipman, Ganbold Lundeg, Kathryn Maitland, Kamal Osman Mergani, Christopher Moschides, Miriam Nakalembe, Ikenna Kingsley Ndu, Jolene Oon, Trina Sale, Ashis Shresthra, Simon Stockley, Daniel Talmor, Audrey Bree Tse, Anand Zachariah, Gavin Matthew Joynt

**Affiliations:** 1 Faculty of Medicine, The Chinese University of Hong Kong, Hong Kong SAR, China; 2 Department of Anaesthesia & Intensive Care, The Chinese University of Hong Kong, Hong Kong SAR, China; 3 Charité Universitätsmedizin, Berlin, 13353 Germany; 4 Department of Anaesthesia, Guy’s and St Thomas’ NHS Foundation Trust, London, UK; 5 Department of Internal Medicine, Saint Paul Hospital, Millennium Medical College, Addis Ababa, Ethiopia; 6 Department of Child Health, Komfo Anokye Teaching Hospital, Kumasi, Ghana; 7 Department of Critical Care Medicine, Hospital Israelita Albert Einstein, Sao Paulo, Brazil; 8 Department of Anesthesia, Critical Care and Emergency Medicine, University of Rwanda, Kigali, Rwanda; 9 Department of Medicine, Brighton and Sussex Medical School, Brighton, UK; 10 World Health Organization, Avenue de Appia 20, Geneva, 1202 Switzerland; 11 Medical Intensive Care Unit, Peking Union Medical College Hospital, Beijing, China; 12 Department of Pathology and Laboratory Medicine, University of British Columbia, BC Children’s Hospital, Vancouver, Canada; 13 Emergency Medicine and Critical Care Medicine, Adults Intensive Care Unit, Hospital Juárez de México, Mexico City, México; 14 Accident and Emergency Medicine Academic Unit, The Chinese University of Hong Kong, Hong Kong SAR, China; 15 Emergency Department,, Hospital de Clinicas, Asunción, Paraguay; 16 Department of Clinical Sciences, Liverpool School of Tropical Medicine, Pembroke Place, Liverpool, UK; 17 ANU Medical School, Canberra Hospital, Woden, Australia; 18 Department of Pediatrics, University of British Columbia, Vancouver, Canada; 19 Royal Brisbane and Womens Hospital and The University of Queensland, Brisbane, QLD Australia; 20 Nimes University Hospital, University of Montpellier, Nimes, France; 21 Department of Critical Care and Anaesthesiology, School of Medicine, Mongolian National University of Medical Sciences, Ulaan Batar, Mongolia; 22 Department of Medicine, St Mary’s Campus, Imperial College London, Norfolk Place, London, UK; 23 KEMRI Wellcome Trust Research Programme, Kilifi, Kenya; 24 Adult Critical Care Medicine Department, Military Hospital, Khartoum, Sudan; 25 Dunvegan Medical Centre, 48 Dunvegan Avenue, Edenvale, Gauging South Africa; 26 Department of Obstetrics and Gynaecology, Makerere University College of Health Sciences, Kampala, Uganda; 27 Department of Paediatrics, College of Medicine, Enugu State University of Science and Technology, Park Lane, Enugu, Nigeria; 28 Division of Infectious Diseases, Department of Medicine, National University Hospital Yong Loo Lin School of Medicine, National University of Singapore, Singapore, Singapore; 29 Emergency Department, National Referral Hospital, Honiara, Solomon Islands; 30 Department of General Practice and Emergency Medicine, Patan Academy of Health Sciences, Patan, Nepal; 31 Eaglescliffe Health Centre, Sunningdale Drive, Eaglescliffe, Stockton-on-Tees, UK; 32 Department of Anesthesia, Critical Care and Pain Medicine, Beth Israel Deaconess Medical Center and Harvard Medical School, Boston, MA USA; 33 Department of Emergency Medicine, John Muir Health, Walnut Creek/ Concord, CA USA; 34 Department of Medicine, Christian Medical College, Vellore, India

**Keywords:** Severe sepsis, Septic shock, Education, Medical students, Competency, Curriculum

## Abstract

**Background:**

Sepsis is a life-threatening condition which may arise from infection in any organ system and requires early recognition and management. Healthcare professionals working in any specialty may need to manage patients with sepsis. Educating medical students about this condition may be an effective way to ensure all future doctors have sufficient ability to diagnose and treat septic patients. However, there is currently no consensus on what competencies medical students should achieve regarding sepsis recognition and treatment. This study aims to outline what sepsis-related competencies medical students should achieve by the end of their medical student training in both high or upper-middle incomes countries/regions and in low or lower-middle income countries/regions.

**Methods:**

Two separate panels from high or upper-middle income and low or lower-middle income countries/regions participated in a Delphi method to suggest and rank sepsis competencies for medical students. Each panel consisted of 13–18 key stakeholders of medical education and doctors in specialties where sepsis is a common problem (both specialists and trainees). Panelists came from all continents, except Antarctica.

**Results:**

The panels reached consensus on 38 essential sepsis competencies in low or lower-middle income countries/regions and 33 in high or upper-middle incomes countries/regions. These include competencies such as definition of sepsis and septic shock and urgency of antibiotic treatment. In the low or lower-middle income countries/regions group, consensus was also achieved for competencies ranked as very important, and was achieved in 4/5 competencies rated as moderately important. In the high or upper-middle incomes countries/regions group, consensus was achieved in 41/57 competencies rated as very important but only 6/11 competencies rated as moderately important.

**Conclusion:**

Medical schools should consider developing curricula to address essential competencies, as a minimum, but also consider addressing competencies rated as very or moderately important.

**Supplementary Information:**

The online version contains supplementary material available at 10.1186/s12909-024-05525-9.

## Introduction

Sepsis is characterized by life-threatening organ dysfunction resulting from the body’s response to infection, estimated to affect 48.9 million patients and account for 11 million deaths each year [1]. The emergence of the coronavirus disease 2019 (COVID-19) pandemic also accentuates the disease burden of sepsis, as sepsis is a frequent complication resulting in high morbidity and mortality [2]. Early recognition and treatment is vital to increase survival and reduce morbidity. As sepsis may result from infection in any organ system, healthcare professionals working in any specialty may need to manage septic patients [3]. Thus, improving survival from sepsis requires all healthcare workers to be educated in its recognition and management.

In 2017 the World Health Assembly adopted a resolution to improve the prevention, diagnosis and management of sepsis and suggested educational development for health care workers [3]. More recently, the development of a core competency framework on sepsis for health care workers was identified as a priority at a WHO Sepsis Technical Expert Meeting [4]. Teaching medical students about sepsis may be an effective way of ensuring that all future doctors have sufficient ability to diagnose and treat septic patients. Medical school curricula are increasingly designed using the competency-based medical education framework [5]. However, there is currently no consensus on what competencies medical students should achieve regarding sepsis recognition and treatment. Determining appropriate competencies is a crucial first step for development of appropriate curricula and for evaluating adequacy of training. Although previous studies have investigated the knowledge of medical students and junior doctors regarding sepsis, the usefulness of these data are limited by the absence of external criteria to judge adequacy [6–9]. Furthermore, a MedLine search using the medical subject headings “sepsis” and “students, medical” and “curriculum” revealed no publications that addressed the issue of what should be included in medical student sepsis curricula.

Most studies of sepsis and recent management guidelines have focused on high income countries/regions but most of the worlds’ population lives in low and middle income countries/regions [10]. In 2017 85% of sepsis cases occurred in areas with low, low-middle or middle standard development index (SDI). Furthermore, the highest age-standardised sepsis-related mortality occurred in areas with the lowest SDI, highlighting the high burden of sepsis in low and middle income countries/regions [1]. The challenges to providing high quality sepsis care likely differ substantially between high income and low/ middle income healthcare systems. For example, 15% of Latin American intensivists reported inadequate conditions to manage patients with septic shock due to insufficient technology, laboratory support, imaging and drug resources [11]. In Africa, only 1.5% of respondents to a survey in 2011, felt they had resources to implement the entire Surviving Sepsis Campaign guidelines [12]. Consequently, it is likely that training requirements for medical students should also differ [9].

We therefore carried out a study, using a Delphi technique, to determine what sepsis-related competencies medical students should achieve by the end of their medical student training in both high or upper-middle incomes countries/regions (HUMIC) and in low or lower-middle income countries/regions (LLMIC) [12]. This study is intended to be the first step in a multi-stage process. Obtaining consensus on what should be included in a sepsis medical school curriculum is the first stage. Further work and studies are required to design and implement a curriculum, develop assessment tools and validate the competencies/ curriculum suggested by this study.

## Method

Approval to carry out the study was obtained from the Survey and Behavioral Research Ethics Committee of The Chinese University of Hong Kong (SBRE 221-17).

An initial search of EMBASE and PubMed using the medical subject headings “sepsis” and “students, medical” and “curriculum” was conducted to identify studies for determining medical student sepsis curricula. No studies were found.

A Delphi method was used to determine sepsis-related competencies which medical students should have achieved by the end of their undergraduate training. The Delphi process involved several stages [13]. Firstly, panels of experts were assembled: one consisting of panelists from HUMIC (as defined by the World Bank for the 2018 fiscal year) and one consisting of panelists from LLMIC [14]. When forming the panel the following factors were considered. First, there should be a balance between panelists with and without a specific interest in sepsis. Second, panelists should come from a range of geographical areas and specialties that commonly manage patients with sepsis. Third, the panel should include doctors who had recently qualified and not yet completed specialist training. Finally, the panel should include members with responsibility for the overall medical school curriculum at their institution. Medical students were not included as panelists as they were deemed to lack sufficient experience and insight regarding sepsis education. However, we believe that recent graduates retain an appreciation of the scope and volume of the whole medical curriculum while understanding the aspects of medical education necessary for patient care. Digital written informed consent to participate was given by all panelists, who volunteered their time without funding.

During the second stage, panelists were asked to individually suggest a broad list of up to 20 competencies that they thought final year medical students should have regarding sepsis. From these suggestions a list of competencies from each of the two panels was compiled. Two researchers (GELM and LL) then individually eliminated duplicates from these lists, with disagreement between the two researchers being resolved by discussion.

During the third stage, the lists of competencies were circulated to the respective panelists, who independently rated the importance of each competency, without awareness of other participant’s ratings, on a numeric scale of 1 to 5 as essential (1), very important (2), moderately important (3), slightly important (4) or unimportant (5) [15].

During the fourth stage, the collated results for each panel were sent to all members of that panel. Panelists were shown their own rating and the distribution of ratings by all other panelists for each competency. Participants were able to change their rating with the understanding that the purpose of the round was to achieve consensus. Consensus was defined as ≥ 75% of participants rating the importance within two adjacent categories; e.g. 75% of participants rating a competency as either essential or very important [16]. After this fourth stage the results were examined by three investigators and an independent adjudicator to determine whether discussion of the precise wording of the competency followed by a further round of voting was likely to result in consensus. This process was completed by 9^th^ May 2020. To facilitate translation and adoption into medical school curricula, competencies were classified according to the 8 commonly accepted competency domains in competency-based medical education: patient care, knowledge for practice, practice-based learning and improvement, interpersonal and communication skills, professionalism, systems-based practice, interprofessional collaboration, and personal and professional development [17]. Median was used to summarize importance ratings.

Submission of suggestions, rating of competencies and revision of ratings were all carried out electronically using REDCap electronic data capture tools hosted at the Department of Anaesthesia & Intensive Care, The Chinese University of Hong Kong [18, 19]. There was no direct face to face discussion between panelists, who all worked independently.

## Results

We invited 19 participants from HUMIC and 15 participants from LLMIC. All HUMIC invitees accepted but one did not respond to any further correspondence leaving 18 panelists. One of the LLMIC invitees did not respond to our invitation and one accepted the invitation but did not respond to any further correspondence, leaving 13 panelists. In the HUMIC group, 14 panelists were from high income countries/regions and four from upper-middle income countries/regions: six from North America, one from South America, four from Asia, four from Europe, two from Oceania and one from Africa. In the LLMIC group, five were from low-income countries/regions and eight from lower-middle income countries/regions: one from North America, three from Asia, one from Oceania and eight from Africa. The specialty areas for which the panelists were invited are given in Table 1. Members of the panel included representatives from World Health Organization (WHO), Global Sepsis Alliance, Latin American Institute of Sepsis, Chinese Society of Critical Care Medicine, African Sepsis Alliance, Asia Pacific Sepsis Alliance, and panelists with overall responsibility for medical school curricula.

**Table 1:** Number of participants representing various specialties in high income and low/ middle income countries/regions

Low and lower middle income countries/regions		High and upper middle income countries/regions	
No. of participants	Specialty area	No. of participants	Specialty area
3	Emergency Medicine	3	Emergency Medicine
2	Intensive Care	6	Intensive Care
3	Pediatrics	1	Pediatrics
2	Infectious diseases	2	Infectious diseases
1	Trainee (Anesthesia)	2	Trainee (Acute Care and Intensive Care)
1	Obstetrics	2	General practice
1	Curriculum management	2	Curriculum management
		1	Pediatric intensive care
		1	World Health Organization

Distribution of specialties represented by participants. Some participants represent multiple specialties, thus the sum is greater than the total number of participants

Panelists in the HUMIC group made 239 suggestions, which were reduced to 109 after removing duplicates. Panelists in the LLMIC group suggested 195, which were subsequently reduced to 88.

After two rounds of rating, consensus was reached for all but one competency in the LLMIC group and all competencies with a median rating greater than very important in the HUMIC group (Table 2). Examination of the distribution of the ratings (by JGM, GCD, LL and GELM) for those competencies for which consensus was not achieved (Supplementary Table 1) suggested that re-wording of the competency was unlikely to achieve consensus if subjected to another round of rating. Competencies rated below moderately important are given in Supplementary Table 2. There was no missing data.

**Table 2:** Competencies rated moderately important or above with consensus

Median rating of importance (1 = essential, 2 = very important, 3 = moderately important	Low and lower-middle income countries/regions	High and upper middle-income countries/regions	Competency Domain
1	**Definitions**		
	Know the definition of sepsis		B
	Know the definition of septic shock		B
		Know the definition of hypotension	B
	**Epidemiology**		
		Know that sepsis can occur in any infected patient	B
		Know the common organisms that cause sepsis (bacteria, viral, fungal, parasites)	B
	**Clinical features and assessment**		
	Know that early recognition of sepsis is important		B
	Know the features of organ dysfunction		B
	Know how to diagnose malaria		A
	Be able to recognize sepsis and septic shock at the bedside	Be able to rapidly clinically assess patients for signs of sepsis	A
	Be able recognize deteriorating respiratory, haematological, hepatic, cardiovascular, central nervous system and renal function by clinical examination		A
	Be able to recognize signs of shock and poor perfusion including tachycardia, hypotension, narrow pulse pressure, clammy peripheries, delayed capillary refill, raised lactate		A
	Be able to take appropriate history and perform clinical examination to identify aetiology of infection and sepsis		A
	Be able to interpret vital signs, including temperature, respiratory rate, blood pressure, pulse, pulse oximetry		A
		Be able to measure body temperature	A
	Be able to examine a child for possible causes of sepsis, including ear, throat and skin examination		A
	Be able to recognize pallor, cyanosis or ashen colour		A
	Be able to measure blood pressure	Be able to measure vital signs including Glasgow Coma Scale, blood pressure, heart rate, respiratory rate	A
	Be able to recognize when local infection becomes sepsis		A
		Be able to recognize a rapidly deteriorating patient	A
	**Investigations**		
	Know the indications for, timing of and how to obtain microbiological cultures		A
	Know what diagnostic tests should be used to rule in or rule out sepsis		B
	Be able to check blood sugar level and interpret results		A
	Be able to interpret the results of investigations to form a diagnosis, identify source of infection and assess severity of organ dysfunction		A
	Be able to interpret complete blood count		A
	Be able to interpret urea, creatinine and electrolyte results		A
		Be able to take venous blood samples properly	A
	**Infection control**		
	Know and follow specific infection control protocols appropriate for the patient and healthcare setting		A, F
		Know when to use and be able to perform aseptic technique properly	A
		Know that infections can be prevented by improved infection control measures and vaccinations	B, F
	Be able to perform hand hygiene properly		A
		Be able to glove and gown properly	A
		Be able to don and remove personal protective equipment properly	A
	**Management**		
	Know that sepsis is an emergency and rapid team-based management is required		F
	Know that antibiotics should be started urgently in sepsis, preferably after appropriate cultures have been taken		B
	Know how to prioritize patients for advanced critical care based on objective parameters when advanced critical care resources are scarce		A
	Know the initial management of septic shock	Know the goals of sepsis management (eg early recognition, timely microbiological investigations and appropriate antibiotics, immediate resuscitation, urgent source control)	B
	Know the targets of resuscitation		B
	Know how and when to judge response to resuscitation and treatment of sepsis		A
		Know that broad spectrum antibiotics should be given initially to target the suspected organisms with guidance from local antibiograms/ guidelines	B
	Know the importance of and how to clean a wound		A
		Know when to intervene to support the airway	B
		Know how to initiate oxygen therapy	B
	Know when to use vasoactive drugs		B
	Be able to initiate treatment with vasoactive drugs		A
	Be able to identify and refer patients outside the hospital who have suspected sepsis for emergency care		A
	Be able to deliver the initial bundle of care (oxygen, antibiotics, fluid resuscitation, monitoring)		A
	Be able to initiate fluid resuscitation	Be able to set up and start an intravenous infusion	A
	Understand that care should focus on the whole patient not an organ or laboratory result		A
	Be able to treat hypoxaemia	Be able to identify early acute respiratory failure and know the possible treatment options	A
	Be able to insert a peripheral venous cannula		A
		Be able to perform basic resuscitation skills including opening the airway and bag-mask ventilation	A
	**Communication**		
	Be able to communicate effectively with the patient and his/her family regarding condition and process of care		D, E
		Be able to communicate concern regarding suspected sepsis and a deteriorating patient	D
	**Miscellaneous**		
		Be able to document findings in medical records	D
1.5	**Epidemiology**		
		Know the most common foci of infection in children and adults	B
	**Clinical features and assessment**		
		Be able to recognize the symptoms and signs of common infections	A
2	**Definition**		
	Know rationale to justify revising definition of sepsis from previous SIRS based definition		B
	Know the SIRS criteria and recognize them when present in a patient		A
	**Epidemiology**		
	Know which patient’s conditions, behaviours, occupations, healthcare settings and age groups increase the risk of sepsis		B
	Know the mortality of sepsis		B
	Know the local epidemiology of antimicrobial susceptibility patterns		B
	Know the common organisms that cause sepsis (bacteria, viral, fungal, parasites)		B
	Know the important comorbidities that contribute to sepsis in your setting		B
	Know the difference between *P. falciparum* and all other malarias in terms of prognosis		B
	**Pathophysiology**		
	Know the most common foci of infection in children and adults		B
	Know the possible sequelae of sepsis in survivors		B
	Know factors that affect capillary refill		B
		Know that sepsis is the final common pathway to death from most infectious disease	B
	Be able to explain the pathophysiology of sepsis		B, D
	**Clinical features and assessment**		
	Know the differential diagnosis of sepsis in the setting where you work		B
	Know how dengue presents differently to other causes of sepsis		B
	Know how to calculate urine output		A
		Know that rash, crepitus and very rapid clinical deterioration are suggestive of sepsis	B
		Know which vital parameters need to be monitored and how frequently	B
		Know that sepsis without finding a definite source of infection or positive microbiological results is common	B
		Know that patients with neutropaenic sepsis may not have an obvious focus of infection	B
		Know the clinical features of uncommon but life-threatening sepsis	B
		Know the situations likely to confound diagnosis or where higher suspicion is required, e.g. pregnancy, immunosuppression	B
		Know that not all signs will be present simultaneously	B
		Know sepsis can still occur when SIRS and qSOFA criteria are not met	B
	Be able to recognize sepsis in neonates, elderly, malnourished and immunocompromised patients		A
	Be able to analyse risk factors to reduce the chance of recurrence		A
	Be able to use a locally used early warning score to follow the patient’s clinical condition		A
		Be able to recognize the need for invasive monitoring of urine output	A
		Be able to clinically evaluate severity of respiratory failure	A
		Be able to identify pathologies amenable to source control	A
	**Investigations**		
	Know the indications and timing of diagnostic laboratory and imaging investigations in a patient with suspected sepsis		A
		Know what additional investigations are indicated to identify the source of sepsis	A
	Know that laboratory investigations should be tailored to the system identified to be affected		A
	Know that lactate should be measured in first 3 h	Know the causes of hyperlactaemia and the concentration that should raise concern	B
	Know how to calculate and utilize qSOFA score		A
		Know the indications for, timing of and how to obtain microbiological cultures	A
	Be able to perform and interpret microbiological investigations		A
	Be able to interpret arterial/venous blood gas results		A
	Be able to interpret blood results (C-reactive protein, procalcitonin, white cell count) in the context of infection and sepsis		A
	Be able to interpret serum lactate result		A
		Be able to perform arterial/venous blood gas sampling	A
		Be able to perform blood cultures using an aseptic technique and inoculating an optimal volume of blood	A
		Know that temperature above 38°C is a sensitive but not specific sign of sepsis	B
	**Management**		
	Know how much initial fluid bolus to deliver for septic shock		B
	Know the potential adverse events of administering fluids in septic patients		B
	Know about the possible adverse consequences of fluid resuscitation in low income countries/regions		B
		Know the indications for fluid resuscitation	B
	Know evidence base to support current recommendations of haemoglobin threshold for transfusion		B
	Know the different routes of parenteral administration of fluids and drugs (peripheral intravenous, central intravenous, intraosseous		B
		Know that at least 2 large bore intravenous access should be established in patients with septic shock	B
	Know the pharmacology and choice of vasoactive drugs		B
		Know when to use vasoactive drugs	B
	Know how to access guidelines for sepsis and up to date information		B
	Know when to use prophylactic antibiotics		B
	Know when and how to de-escalate antibiotic therapy	Know that antimicrobial choice and duration should be reviewed daily	B
		Know that empirical antibiotics should be stopped if patients don’t have infection	B
		Understand the importance of antimicrobial resistance and its implications for sepsis management	B
	Knows the evidence to support the current recommendations for paediatric shock		B
		Know when escalation of care might not be appropriate (eg extreme frailty)	B
		Know the appropriate management of indwelling devices to reduce the risk of sepsis	B
		Know the targets of resuscitation	B
		Know how and when to judge response to resuscitation and treatment of sepsis	B
	Be able to identify and refer for an appropriate level of care		A
	Be able to insert urinary catheter properly		A
	Know the anatomical landmarks and be able to insert an intraosseous needle		A
	Be able to explain the principles of early goal directed therapy		B, D
	Be able to identify the most appropriate means to transport a patient with suspected sepsis for emergency care		A, G
	**Miscellaneous**		
	Be able to train staff on what to do next and properly document the care plan		D, G
		Describe appropriate safety netting relevant to sepsis	B
		Know how to update health records with infection information at transitions of care	D
		Be able to take consent appropriately	D, E
3	**Pathophysiology**		
		Explain the pathophysiology of sepsis	B
		Know the metabolic derangement that occurs with sepsis	B
	**Clinical features and assessment**		
		Distinguish between community and hospital acquired sepsis	A
	**Investigations**		
	Know how can bedside ultrasound be used to monitor fluid resuscitation in patients		B
	Be able to perform arterial/venous blood gas sampling		A
	**Management**		
	Know the steroids may play a role in treatment of sepsis		B
		Know when and how to de-escalate antibiotic therapy	B
	**Miscellaneous**		
	Know about the cost of treatment		B
		Know the possible sequelae of sepsis in survivors	B

This table shows the competencies with a median rating of moderately important or above for which consensus was achieved. Competencies that are identical in the two groups span the columns and competencies that are similar but not identical are given in the same row but different columns. Competencies were classified into 8 domains: patient care (A), knowledge for practice (B), practice-based learning and improvement (C), interpersonal and communication skills (D), professionalism (E), systems-based practice (F), interprofessional collaboration (G) and personal and professional development (H) [16]

*SIRS* Systemic inflammatory response syndrome, *qSOFA* Quick Sepsis Related Organ Failure Assessment

## Discussion

Our study is the first study to identify sepsis-related competencies that medical students should achieve by graduation. This is in line with the priority identified at the WHO Sepsis Expert Technical meeting in 2018 to develop core competency frameworks on sepsis for health care workers. We identified 38 and 33 essential competencies which medical students should achieve in LLMIC and HUMIC, respectively. Consensus was reached for all essential competencies. In the LLMIC group, complete consensus was also achieved for competencies ranked as very important and was achieved in 4/5 competencies rated as moderately important. In the HUMIC group, consensus was achieved in 41/57 competencies rated as very important but only 6/11 competencies rated as moderately important. These competencies have been endorsed by the Global Sepsis Alliance to guide development of sepsis curricula for medical students.

Given the burden of sepsis and the need for early intervention, doctors working in all specialties should have the basic competencies required for diagnosing and managing septic patients. It therefore behoves medical schools to provide training in sepsis recognition and management. Indeed, it has been suggested that member states of the WHO should mandate this training for all healthcare workers [4].

We believe the competencies identified in this study provides a useful framework on which to develop sepsis training for medical students. The aim of modern competency-based medical education is to train “health-professionals that can practice medicine at a defined level of proficiency” [20]. Most of the identified competencies were within the domains of patient care, knowledge for practice and interpersonal and communication skills [17]. This reflects the objective of this study which was to identify core competencies that medical students should achieve by graduation to care for patients with sepsis.

While we understand that different medical schools will have different priorities for teaching, we strongly suggest that, as a minimum, training to achieve the competencies rated essential be incorporated into the curriculum of all medical schools. We also suggest that medical students should achieve competencies rated very important or moderately important for which consensus was obtained (Table 2). For example, both LLMIC and HUMIC panels ranked “know the definition of sepsis” and “know that early recognition of sepsis is important” as essential competencies. Meanwhile, “know the SIRS criteria and recognize them when present in a patient”, “know how to calculate and utilize qSOFA score” and “know rationale to justify revising definitions of sepsis from previous SIRS based definition” were only ranked as very important rather than essential. Whilst these seemingly similar competencies resulted in different importance rankings, the need to know the definition of sepsis and importance of early sepsis recognition are key concepts of sepsis management. Instead, SIRS or qSOFA criteria are imperfect tools for sepsis recognition which may be updated or replaced over time. Indeed, differences in use of SIRS and qSOFA to diagnose and prognosticate sepsis when compared to Sepsis-3 criteria based on SOFA has been well documented [21]. This distinction in ranking may help medical schools prioritize essential learning objectives in curriculum development.

A guiding principle of competency based-medical education is to achieve competencies that are “in accord with local conditions to meet local needs [20]”. We anticipated that LLMIC would have different priorities and requirements for medical students compared with HUMIC. Therefore, LLMIC and HUMIC had separate panels and thus separate results. While many of the suggested competencies overlapped, there were some suggestions that differed between the groups (Table 2 and Supplementary Table 1). For example, an essential competency for LLMIC is to know how to diagnose malaria, while this was not even suggested HUMIC group. In addition, some basic skills such as being able to measure blood pressure and blood glucose were included by the LLMIC panel but not the HUMIC panel. Furthermore, an essential competency included by the LLMIC but not HUMIC panel was to be able to prioritize patients needing critical care when resources are scarce. While we attempted to address heterogeneity by having separate LLMIC and HUMIC panels, this does not address the heterogeneity in diseases and resources within LLMIC or HUMIC.

Studies based on Delphi methodology are entirely dependent on the opinion of their expert panels [22]. By their very nature these panels are not randomly selected and the selection process may introduce bias. We used specific criteria to determine the composition of the panels to minimize bias. In particular, we deliberately included panelists who did not have a particular interest in sepsis and panelists whose professional position allowed them an overview of their entire medical school curriculum. There was, perhaps, an over-representation of Intensive Care specialists in the HUMIC group, but the inclusion of life-threatening organ dysfunction in sepsis criteria means that Intensive Care specialists are inevitably major stakeholders. Furthermore, the relatively high proportion of Intensive Care specialists on the panel does not appear to have been reflected in those competencies considered essential, none of which are specific to Intensive Care practice. In retrospect, the failure to include public health specialists was a weakness, which may have resulted in an absence of suggestions for public health interventions such as vaccination.

We defined consensus as 75% of panelists rating the importance of a competency within two adjacent categories. Percentage agreement is the most common method of defining consensus and the median threshold for agreement in 25 Delphi studies that were recently reviewed was 75%, with a reported threshold agreement range of 50–97% [16]. There is no consensus on the number of panelists required, but our number of panelists falls within the commonly used range. In a review of 76 healthcare related Delphi studies the median number of individuals invited to participate was 17 (interquartile range of 11–31), and in a review of 100 studies the most common number of panelists in the final round was between 11 and 25 [23, 24].

While the Delphi method is a widely used method for developing guidelines and curricula, there are no published data to demonstrate that Delphi-based curricula improve students’ learning outcomes. This is partly due to difficulties in separating the effects of curriculum and curriculum content.

Another potential limitation is that panelists didn’t meet to discuss results. It is possible that consensus wasn’t achieved for some competencies because panelists weren’t able to discuss the reasoning behind their suggestions. In contrast, an advantage of the panelists not meeting was the absence of social pressure to agree with a suggestion that they fundamentally did not agree with, thus avoiding false consensus.

It is possible that greater consensus would have been achieved if a further round of rating had been undertaken after discussion on re-wording competency statements. However, after careful assessment, the adjudicating group believed that this was unlikely, and that considering the level and nature of competencies for which consensus was not reached, greater consensus would not substantially increase the usefulness of this document.

The results of this study were collected prior to the 2021 update to the Surviving Sepsis Campaign International Guidelines for the management of sepsis. While it is possible that these newer guidelines might have altered the panelists’ recommendations, the relatively non-specific nature of the recommendations mean it is unlikely that they would be significantly impacted by changes in guidelines regarding the specific management of sepsis [25].

As mentioned above, the results of the study are based on expert opinion not high level evidence. However, we are not aware of any high level evidence on which to base a medical student sepsis curriculum. Once a curriculum and assessment based on these competencies has been developed, further research to validate our data as a tool to strengthen medical students’ experience and clinical performance may be possible.

## Conclusion

We have identified essential sepsis-related competencies for medical students in both LLMIC and HUMIC. Consensus on their importance was achieved for all these competencies. We suggest that medical schools develop curricula to address these competencies, as a minimum, but also consider addressing competencies rated as very or moderately important.

### Supplementary Information

Supplementary Material 1.

## Data Availability

All data generated or analysed during this study are included in this published article [and its supplementary information files].
