# Supplementary material for: Core sepsis-related competencies for medical students: an international consensus by Delphi technique
Source: BMC Med Educ. 2024 Jun 11;24:653. doi: 10.1186/s12909-024-05525-9 (PMC11167876; doi:10.1186/s12909-024-05525-9)
Supplement: Supplementary file 1 — Supplementary Material 1. [file 12909_2024_5525_MOESM1_ESM.docx]

**Supplementary tables**

**Supplementary Table 1 Competencies with a median rating of moderately important or above, where consensus was not achieved.**

| Median rating of importance (1=essential, 2=very important, 3=moderately important) | Low and lower-middle income countries/regions | High and upper-middle income countries/regions | Competency Domain |
| --- | --- | --- | --- |
| 2 | **Definitions** | |  |
|  |  | Know the definition of SIRS | B |
|  | **Epidemiology** | |  |
|  |  | Know that sepsis is a global health burden and the burden relative to other major killers such as cancer and heart diseases | B |
|  |  | Understand that sepsis does not occur only in the healthcare setting by unclean care and is not primarily caused by multi-resistant bacterial super bugs, but that about 80% of sepsis cases develop in the community and often presents as the clinical deterioration of common and preventable infections such as those of the respiratory, gastrointestinal and urinary tract, or of wounds and skin | B |
|  | **Pathophysiology** | |  |
|  |  | Understand the physiology of fluid volume response | B |
|  | **Clinical features and assessment** | |  |
|  |  | Know that patients with SIRS criteria do not necessarily have sepsis and how treatment differs | B |
|  |  | Be able to perform basic evaluation of fluid volume responsiveness | A |
|  | **Investigations** | |  |
|  |  | Know left shift of leucocytes is suggestive of sepsis | B |
|  |  | Know that a raised white cell count above 12 is a sensitive but non-specific sign of sepsis | B |
|  | **Management** | |  |
|  |  | Know the role of antimicrobial stewardship in maximising patient outcomes from sepsis and minimising adverse reactions, antimicrobial resistance and costs | B |
|  |  | Know what target SpO2 is needed to manage patient with shock | B |
|  |  | Know the importance of implementing source control interventions as soon as possible | B |
|  |  | Know the importance of monitoring fluid balance | B |
|  |  | Understand the pharmacology of fluid solutions, how they differ, how to use them and side effects | A |
|  |  | Be able to insert a urinary catheter properly | A |
|  |  | Be able to choose appropriate antibiotics (side effect, cost, administration, activity spectrum, availability) | A |
|  | **Miscellaneous** | |  |
|  |  | Know the importance of and be able to contribute to and utilize the multidisciplinary team effectively | A |
| 3 |  | Know the SIRS criteria and recognize them when present in a patient | A |
|  |  | Know the indications for inserting a central venous catheter in sepsis | B |
|  |  | Know how to measure central venous pressure | B |
|  |  | Know initial ventilator management | B |
|  |  | Describe key performance indicators for sepsis management | B |
|  | Be able to perform abscess drainage, wound debridement, aspirate abdominal fluid and send appropriate specimens for culture |  | A |

The scale of importance ranged from 1=essential, 2=very important, 3=moderately important, 4=slightly important to 5=unimportant. Competencies were classified into 8 domains: patient care (A), knowledge for practice (B), practice-based learning and improvement (C), interpersonal and communication skills (D), professionalism (E), systems-based practice (F), interprofessional collaboration (G) and personal and professional development (H) (16). SIRS, Systemic Inflammatory Response Syndrome.

**Supplementary Table 2 Competencies with a median rating below moderately important from the HUMIC group**

| Median importance | Competency | Competency Domain |
| --- | --- | --- |
| Consensus achieved | |  |
| 4.5 | Be able to do a basic echo exam including recognizing hypovolemia | A |
| Consensus not achieved | |  |
| 3.5 | Be able to perform endotracheal intubation in septic patients with respiratory failure | A |
|  | Know how to perform invasive line insertion (arterial and central venous) | B |
| 4 | Know how to use ultrasound to evaluate for other causes of shock, such as RV failure, LV failure | B |

The scale of importance ranged from 1=essential, 2=very important, 3=moderately important, 4=slightly important to 5=unimportant. No competencies were related below moderately important by the low or lower-middle income group. Competencies were classified into 8 domains: patient care (A), knowledge for practice (B), practice-based learning and improvement (C), interpersonal and communication skills (D), professionalism (E), systems-based practice (F), interprofessional collaboration (G) and personal and professional development (H) (16). LV, left ventricular; RV, right ventricular.
